# Geriatric Fever Score: A New Decision Rule for Geriatric Care

**DOI:** 10.1371/journal.pone.0110927

**Published:** 2014-10-23

**Authors:** Min-Hsien Chung, Chien-Cheng Huang, Si-Chon Vong, Tzu-Meng Yang, Kuo-Tai Chen, Hung-Jung Lin, Jiann-Hwa Chen, Shih-Bin Su, How-Ran Guo, Chien-Chin Hsu

**Affiliations:** 1 Department of Emergency Medicine, Chi-Mei Medical Center, Tainan, Taiwan; 2 Department of Emergency Medicine, Chi-Mei Medical Center, Liouying, Tainan, Taiwan; 3 Department of Environmental and Occupational Health, College of Medicine, National Cheng Kung University, Tainan, Taiwan; 4 Department of Child Care and Education, Southern Taiwan University of Science and Technology, Tainan, Taiwan; 5 Department of Emergency Medicine, Kuo General Hospital, Tainan, Taiwan; 6 Department of Emergency Medicine, Taipei Medical University, Taipei, Taiwan; 7 Department of Biotechnology, Southern Taiwan University of Science and Technology, Tainan, Taiwan; 8 Department of Emergency Medicine, Cathay General Hospital, Taipei, Taiwan; 9 Fu Jen Catholic University School of Medicine, Taipei, Taiwan; 10 Department of Occupational Medicine, Chi-Mei Medical Center, Tainan, Taiwan; 11 Department of Leisure, Recreation and Tourism Management, Southern Taiwan University of Science and Technology, Tainan, Taiwan; 12 Department of Medical Research, Chi Mei Medical Center, Liouying, Tainan, Taiwan; 13 Department of Occupational and Environmental Medicine, National Cheng Kung University Hospital, Tainan, Taiwan; University of Dundee, United Kingdom

## Abstract

**Background:**

Evaluating geriatric patients with fever is time-consuming and challenging. We investigated independent mortality predictors of geriatric patients with fever and developed a prediction rule for emergency care, critical care, and geriatric care physicians to classify patients into mortality risk and disposition groups.

**Materials and Methods:**

Consecutive geriatric patients (≥65 years old) visiting the emergency department (ED) of a university-affiliated medical center between June 1 and July 21, 2010, were enrolled when they met the criteria of fever: a tympanic temperature ≥37.2°C or a baseline temperature elevated ≥1.3°C. Thirty-day mortality was the primary endpoint. Internal validation with bootstrap re-sampling was done.

**Results:**

Three hundred thirty geriatric patients were enrolled. We found three independent mortality predictors: Leukocytosis (WBC >12,000 cells/mm^3^), Severe coma (GCS ≤ 8), and Thrombocytopenia (platelets <150 10^3^/mm^3^) (LST). After assigning weights to each predictor, we developed a Geriatric Fever Score that stratifies patients into two mortality-risk and disposition groups: low (4.0%) (95% CI: 2.3–6.9%): a general ward or treatment in the ED then discharge and high (30.3%) (95% CI: 17.4–47.3%): consider the intensive care unit. The area under the curve for the rule was 0.73.

**Conclusions:**

We found that the Geriatric Fever Score is a simple and rapid rule for predicting 30-day mortality and classifying mortality risk and disposition in geriatric patients with fever, although external validation should be performed to confirm its usefulness in other clinical settings. It might help preserve medical resources for patients in greater need.

## Introduction

The geriatric population (≥65 years old) constituted 6.2% of the world population in 1992 and is expected to reach 20% by 2050 in the world [1] and as early as by 2030 in the United States [2]. In Taiwan, it was 7% in 1993 and reached 11.33% in July 2013 [3]. As the elderly population increases, the need for medical and healthcare resources increases, especially emergency medical care. Older people account for 12–24% of all emergency department (ED) visits [4], and 10% of the elderly paying such visits have a fever, of which 70–90% will be admitted and 7–10% will die within a month [5], [6]. Therefore, fever is worrisome in the geriatric population.

Infectious diseases are the cause of acute fever requiring hospitalization in 75% of geriatric patients [1]. However, 20–30% of the elderly with an infection present to the ED with a blunted fever response, in part because of a lower basal body temperature [7]. Other causes include changes in thermal homeostasis, decreased response to endogenous and exogenous pyrogens, decreased production and conservation of body heat, comorbidities, and drugs [8]. For detecting acute infection using body temperature, while a higher cut-point is more specific, a lower cut-point provides a higher sensitivity, which is more important to avoid missing serious infections [8]. The current consensus is that using a tympanic temperature ≥37.2°C or a baseline temperature elevated ≥1.3°C is most appropriate for defining fever [2], [8], [9].

Evaluating geriatric patients with fever is time-consuming and challenging [8]. The fever may be attributable to an atypical disease, drug effects, or multiple comorbidities [8]. The ED physician always faces two questions: [a] What is the mortality risk for this patient? and [b] What is the most appropriate disposition (viz., discharge, treatment in the ED, admission to a general ward, or admission to the intensive care unit [ICU]) based on the balance of patient safety, cost, and availability of medical resources after ED treatment? Therefore, some studies have proposed decision rules based on mortality predictors to help ED physicians make optimum decisions on how best to manage geriatric patients with fever. However, most of the reported predictors are impractical for the ED. For example, one study [10] proposed that altered mental status, vomiting, and a white blood cell count (WBC) band form >6% were independent predictors of bacteremia. Another [11] proposed that all patients had bacterial infection when they had fever (≥37.5°C), leukocytosis (WBC ≥14,000 cells/mm^3^), and bandemia (band form >6%). A third [12] proposed that an oral temperature ≥39.4°C, a respiratory rate ≥30/min, leukocytosis (WBC ≥11,000 cells/mm^3^), infiltration on a chest radiograph, and a pulse rate >120/min were associated with serious illness. However, altered mental status, vomiting, and infiltration on a chest radiograph are difficult to verify for geriatric patients, and thus these predictors have low reliability among ED physicians. Furthermore, these three studies were conducted 20 years ago, and many factors might be different because of changes in demography and geriatric care. We thus conceived a research of developing a feasible and applicable contemporary prediction rule for decision-making in a hospital ED.

## Methods

### Study design, setting, population, and selection of participants

This study was conducted in a 700-bed university-affiliated medical center in Taipei with a 40-bed ED staffed with board-certified emergency physicians that provides care for approximately 55,000 patients per year. About 33% of the ED patients are elderly. Consecutive geriatric patients who visited the ED between June 1 and July 21, 2010, were enrolled when they met one of the following criteria of fever [2], [8], [9]: a tympanic temperature (TM) ≥37.2°C or a baseline temperature elevated ≥1.3°C. The baseline temperature information came from the previous medical record, patient, caregiver, or nursing home staff.

### Data Collection and Definition of Variables

Patients were prospectively selected in the ED. After the patient had been discharged, reviewers retrospectively collected missing information from the medical record or a telephone follow-up in compliance with the policies approved by Institutional Review Boards (IRBs) at Cathay General Hospital, which also approved the study protocol. The IRBs waived the need for informed consents (written and oral) from the participants because this is an observational study. The reviewers were blinded to knowledge of the patient’s hospital course and outcomes. Information for a number of variables for each patient was recorded (Tables 1 and 2). Any variable not included in the patient’s medical history or physical examination reports was considered missing.

**Table 1:** Patient characteristics of geriatric patients with fever in the Emergency Department.

	Total Patients (n = 330)
Age (mean ± SD)	78.5±7.7
Age subgroup, %	
Young elderly (65–74 years)	33.6
Moderately elderly (75–84 years)	40.6
Old elderly (≥85 years)	25.8
Gender: Male (%)	45.8
Vital signs (mean ± SD)	
Glasgow coma scale	14.0±2.3
Systolic blood pressure (mmHg)	144.0±28.6
Heart rate (n/min)	98.2±21.0
Respiratory rate (n/min)	20.7±3.8
Body temperature (°C)	38.0±0.1
Medical history (%)	
Diabetes	29.1
Hypertension	57.0
Stroke	19.1
Cancer	10.9
Congestive heart failure	8.5
Chronic pulmonary obstructive disease	10.9
Dementia	6.7
Nursing home resident	4.5
Bedridden	19.4
Nasogastric tube feeding	11.5
Laboratory data (mean ± SD)	
White blood cell count (cells/mm^3^)	10600.0±4534.0
Hemoglobin (g/dL)	12.2±2.1
Platelet (10^3^/mm^3^)	186.1±74.0
Serum creatinine (mg/dL)	1.4±1.3
C-reactive protein (mg/dL)*	6.3±7.1
ED diagnosis (%)†	
Urinary tract infection	29.7
Low respiratory tract infection	22.1
Fever without significant focus	7.6
Intra-abdominal infection	7.0
Upper respiratory tract infection	3.6
Skin or soft tissue infection	3.0
Influenza	2.7
Cancer	2.7
Bone/joint infection	1.8
Congestive heart failure	1.8
Sepsis	1.2
Admission rate (%)‡	51.8
30-day mortality rate (%)	6.7

SD, standard deviation; ED, Emergency Department. *286 (86.7%) patients had this test. †Not all the ED diagnoses are listed in the table. ‡Admission to general ward or intensive care unit.

**Table 2:** Univariate mortality predictors at *P*<0.1 of geriatric patients with fever in the Emergency Department.

	Variable Present		
	Yes	No	*P-* value
Variable	n (% mortality)	n (% mortality)	
Severe coma (GCS ≤ 8)	12 (41.7)	318 (5.3)	<0.001
Hypotension (SBP<90 mmHg)	9 (44.4)	321 (5.6)	0.002
Tachypnea (RR >20/min)	96 (10.4)	234 (5.1)	0.091
Stroke history	63 (12.7)	267 (5.2)	0.046
Bedridden	64 (15.6)	266 (4.5)	0.001
Nasogastric tube feeding history	38 (18.4)	292 (5.1)	0.007
Congestive heart failure history	28 (17.9)	302 (5.6)	0.029
Nursing home resident	15 (20.0)	315 (6.0)	0.069
Leukocytosis (WBC >12,000 cells/mm^3^)	111 (11.7)	219 (4.1)	0.017
Thrombocytopenia (Platelets <150×10^3^/mm^3^)	102 (11.8)	228 (4.4)	0.013
Bandemia (>10% band)	5 (40.0)	325 (6.2)	0.038
Serum creatinine >2 mg/dL	38 (21.1)	292 (4.8)	0.001

GCS, Glasgow coma scale; SBP, systolic blood pressure; RR, respiratory rate; WBC, white blood cell count.

The categorical variables used are generally acceptable in emergency care, critical care, and geriatric care. *Severe coma* was defined as a Glasgow Coma Scale (GCS) score ≤8 [13]. *Bedridden* was defined as an Eastern Cooperative Oncology Group (ECOG) Performance Status (also called the WHO or Zubrod score) Score 4: completely disabled, cannot carry on any self-care and totally confined to bed or chair [14]. *Hypotension* was defined as systolic blood pressure <90 mmHg, *and leukocytosis* was defined as a white blood cell (WBC) count >12,000 cells/mm^3^ [15]. *Serum creatinine >2 mg/dL* was a criterion of severe sepsis [15]. *Thrombocytopenia* was defined as a platelet count <150 10^3^/mm^3^ [16]. The infections diagnosed in the ED included urinary tract infection, lower respiratory tract infection, fever without a significant focus, intra-abdominal infection, upper respiratory tract infection, skin or soft tissue infection, influenza, bone/joint infection, and sepsis. The clinical diagnosis was based on the attending physician’s documentation, and on laboratory and image results (such as pneumonia on a chest radiograph, pyuria on urinary analysis, abscess or intracranial hemorrhage on computed tomography, etc). The diagnosis of influenza was based on the clinical practice guidelines of the Infectious Diseases Society of America [17].

**Figure 1:**
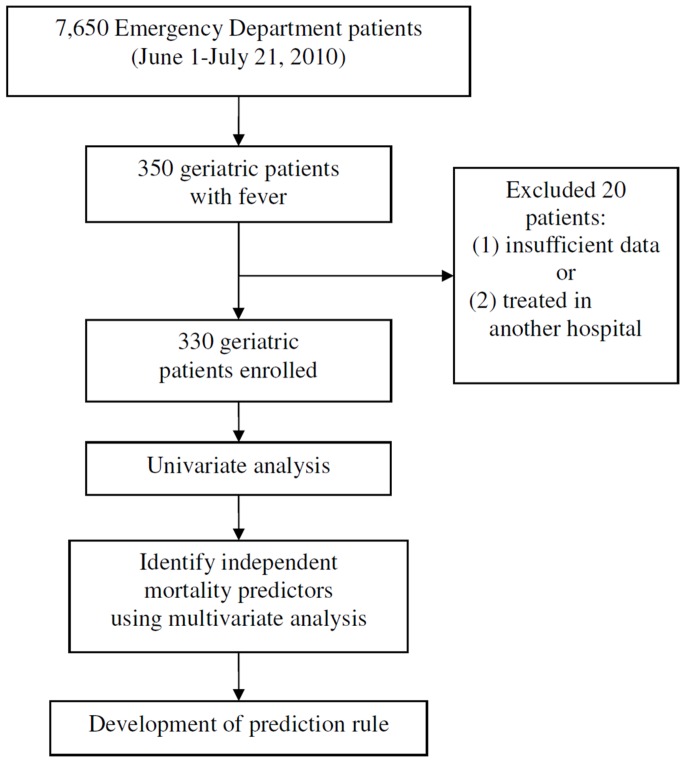
Patient enrollment plus flowchart of how the prediction rule was developed.

Overall, 350 geriatric patients from the ED met the criterion of fever. Three hundred thirty patients were enrolled after excluding 20 patients with insufficient data or transferred patients who had been treated in other hospitals. The enrolled patients were divided into 2 groups based on their 30-day outcome, survival, or mortality. All the study variables were used for comparisons between groups. Figure 1 shows the study flowchart.

### Definition of Endpoint

We used 30-day mortality as the primary endpoint. People who survived at least 30 days were considered “survivors” for this analysis. Telephone follow-up was used to ascertain 30-day survival if discharged before 30days.

### Data analysis

SPSS 20.0 for Windows (Chicago, IL, USA) was used for all statistical analysis. Continuous data are presented as means ± SD. A univariate regression analysis was done using either an independent samples *t* test (assuming a normal distribution) or a Mann-Whitney and Wilcoxon test (assuming non-normality) for the continuous variables. Either a χ^2^ test or a Fisher’s exact test was used for the categorical variables.

The significant α level was set at 0.1 for univariate variables that were included in a multiple logistic regression analysis of risk for 30-day mortality. Significance was set at P<0.05 (2-tailed) to extract variables effective in a model. If a case is missing data for any of the variables in the analysis, it will be dropped entirely from the model. The area under the receiver operating characteristic (ROC) curves was used to compare a model's specifications.

The results of the multivariate stepwise (forward) logistic regression analysis were then used to develop a clinical prediction rule [18]. Weights were assigned to each predictor according to the predicted β values of the multiple logistic regression analysis. Each β coefficient was divided by 2 and rounded to the nearest integer. A Geriatric Fever Score was then calculated for each patient. We used bootstrapping methods to assess the stability of the score. Using random sampling from actual study patients, we generated 1,000 hypothetical study populations. We estimated coefficient point estimates with the reduced model for each hypothetical population. We estimated the bootstrapped effect size and 95% confidence interval [CI]s for each coefficient. Receiver operating characteristics curve and area under the receiver operating characteristics curve were generated with bootstrap methods. The 95% CIs of the area under the receiver operating characteristics curve and differences in area under the receiver operating characteristics curve between the weighting schemes were obtained according to 1,000 bootstrap samples.

## Results

We enrolled 330 geriatric patients, about 4.3% of all patients who visited the ED during the study period. Admission and 30-day mortality rates were 51.8% and 6.7%, respectively (Table 1). The most common causes of fever were urinary tract infection (29.7%), lower respiratory tract infection (22.1%), fever without significant focus (7.6%), intra-abdominal infection (7.0%), and upper respiratory tract infection (3.6%) (Table 1). Twelve univariate mortality predictors were at the criterion of *P*<0.1 (Table 2); body temperature >38°C did not appear to be a significant predictor (*P* = 0.205). After a multiple logistic regression analysis, three independent mortality predictors were identified: **L**eukocytosis, **S**evere coma, and **T**hrombocytopenia (LST pronounced as LiST) (Table 3). Age itself and age subgroups did not predict the prognosis. We used the independent mortality predictors to assign point values for a decision rule-Geriatric Fever Score (Table 3).

**Table 3:** Independent mortality predictors identified using multivariate analysis of geriatric patients with fever in the Emergency Department.

	Parameters			Geriatric Fever
Variable	β	Bootstrapped OR	Bootstrapped 95% CI	Score Points
Intercept	−4.967			
***L***eukocytosis (WBC >12,000 cells/mm^3^)	1.488	4.4	1.5–13.0	1
***S***evere coma (GCS ≤ 8)	2.330	10.3	1.9–54.7	1
***T***hrombocytopenia (Platelets <150×10^3^/mm^3^)	1.305	3.7	1.2–11.0	1
AUC				0.73
Possible scores in a range				0–3

OR, odds ratio; CI, confidence interval; GCS, Glasgow coma scale; AUC, area under the curve.

**Figure 2:**
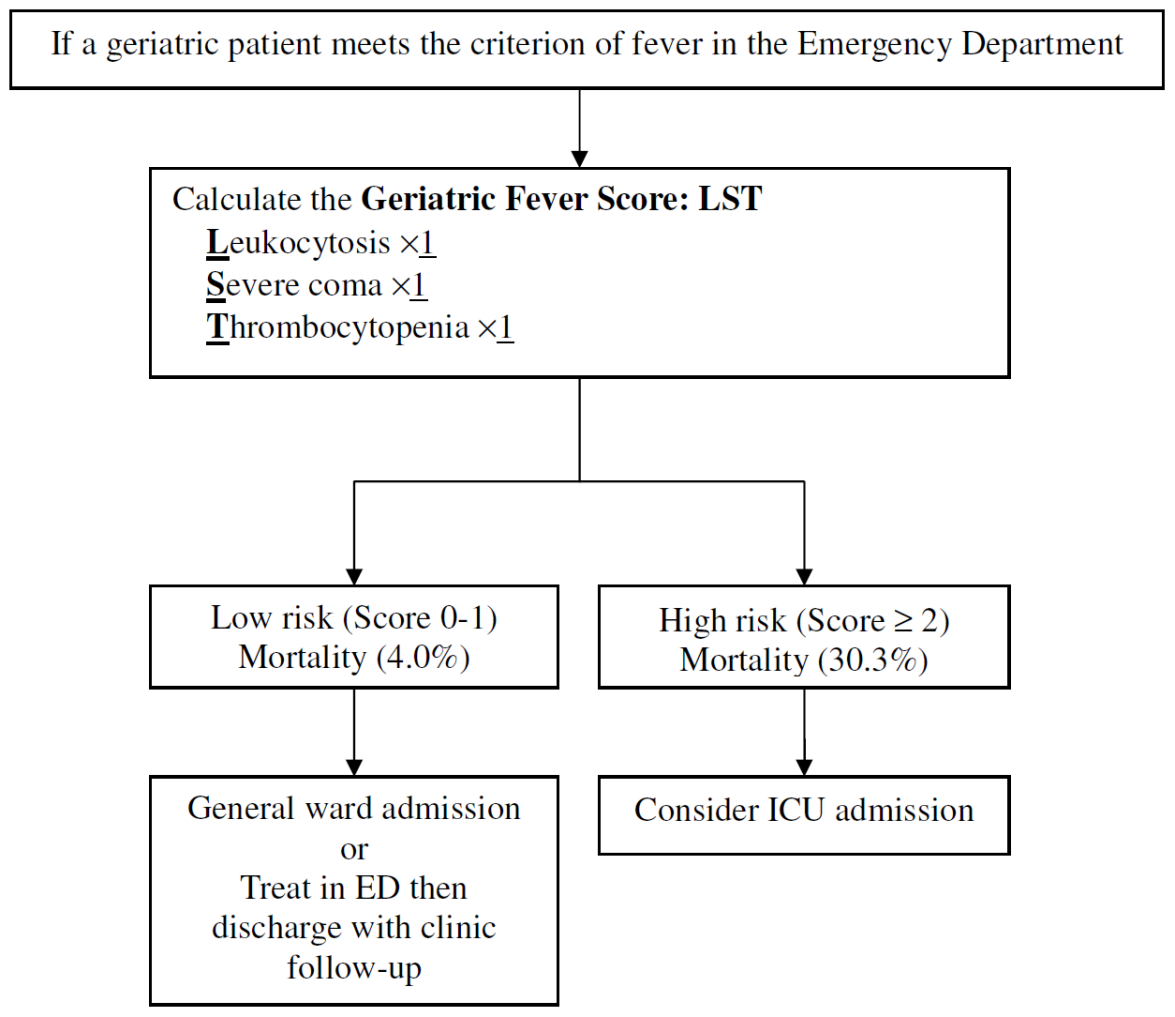
A suggested disposition flowchart based on the Geriatric Fever Score for geriatric patients with fever in the emergency department (ED).

When the Geriatric Fever Score was applied, it stratified patients into two risk groups with very different mortality rates: low risk = 4.0% (12/297, 95% CI: 2.3–6.9%) and high risk = 30.3% (10/33, 95% CI: 17.4–47.3%) (Figure 2). The mortality by the Geriatric Fever Score is 2.9% (4/140) for patients with a score of 0, 5.1% (8/157) for 1, 25.8% (8/31) for 2, and 100% (2/2) for 3. Because the mortality was similar in those with scores 0 and 1 and the number of patients with the score 3 was too small (only 2), we use the score 2 as the cut-off to divide the patients into two groups. The area under the ROC curve (Table 3) for the Geriatric Fever Score was 0.73 (95% CI: 0.61–0.85), which showed good diagnostic accuracy. Severe Coma appeared to be a stronger predictor than others; however, the area under the ROC curve was inferior to assigning single point to all predictors (0.729 vs. 0.730) when severe coma was assigned with 2 points. Therefore, we gave all predictors single point for better discrimination and also simplicity.

Sixteen of the 22 patients (72.7%) who died within 30days succumbed to sepsis, 3 patients (13.6%) to sepsis with end-stage cancer, 1 patient (4.5%) to intracranial hemorrhage, 1 patient (4.5%) to upper gastrointestinal bleeding, and 1 (4.5%) patient to an acute coronary syndrome. Totally, 12.5% of the patients with fever died from non-infection related illness.

## Discussion

We developed a novel decision rule to predict 30-day mortality and manage geriatric patients with fever in the ED. Emergency care, critical care, and geriatric care physicians can usefully evaluate three variables. Geriatric patients with a high-risk Geriatric Fever Score should be deemed critically ill and considered to be sent to the ICU for advanced treatment. For patients with a low-risk score, a transfer to a general ward or treatment in the ED then discharge with close follow-up in an outpatient clinic depends on the treating physician's decision based on the each patient's condition and on available medical resources, which help preserve medical resources for patients in greater need.

Multiple logistic regression analysis identified three independent correlates of mortality. Severe coma, with a high β-value, was the strongest predictor of mortality. Geriatric patients who present with an infection may have atypical symptoms, such as altered mental status or a decline in functional physical status, in cognitive status, or in both [2]. However, altered cognitive status is difficult to accurately recognize because it must be compared with each patient’s baseline mental status. In our study, a severe coma instead of altered cognitive status was used as an independent predictor of mortality, which is realistic and is feasible to quantify in clinical practice. Leukocytosis, a criterion of sepsis in Surviving Sepsis Campaign Guidelines 2012 [15], was identified as an independent predictor of mortality in our study. Different studies have proposed different definitions of leukocytosis in geriatric patients. Marco et al. [12], who surveyed geriatric patients presenting with fever in the ED, defined leukocytosis as WBC >11,000 cells/mm^3^ and said that it was predictive of serious illness. Wasserman et al. [11], however, found that increasing leukocyte counts of 11,000, 14,000, or 16,000/mm^3^ were associated with increasing specificity but decreasing sensitivity. Rather than one of these two definitions of leukocytosis, we used WBC >12,000 cells/mm^3^, which is more acceptable and more commonly used [15]. Prior studies had proposed that baseline functional dependence, such as being bedridden, was the most prevalent risk factor predicting various adverse outcomes in elderly patients in the ED [10], [11], [19]. In our study, *bedridden* was defined as an ECOG Score of 4: completely disabled, cannot carry on any self-care and totally confined to bed or chair [14]. The ECOG Score has the advantage of simplicity over other performance status scores such as the Karnofsky scale [20] and the Barthel scale [21]. Thrombocytopenia, a criterion of sepsis [15], was also an independent predictor of mortality in our study. Because the normal range of platelets is 150–400×10^3^/mm^3^, defining thrombocytopenia as platelets <150×10^3^/mm^3^, which we did in our study, should be easier to remember than platelets <100×10^3^/mm^3^, which was used in the Surviving Sepsis Campaign Guidelines 2012 [15].

This study has several limitations. First, some data were collected from a retrospective chart review. The clinical presentations or records may not have been completely documented. Second, this was a single-center study. Findings from our database may not be generalizable to other cohorts in Taiwan or to cohorts in other nations. Third, the sample size might not be large enough to make conclusions with good statistical power. Additional studies with larger samples are necessary. Fourth, despite the fact that we have performed internal validation for the same population, external validation in other populations should also be performed to confirm the usefulness of this score system. Fifth, we did not evaluate some known risk factors such as albumin, increase of leukocyte counts from baseline, lactate, and procalcitonin; whereas they might be useful, it is not practical to perform these tests or obtain the data on every geriatric patient with fever.

## Conclusion

The Geriatric Fever Score is a simple and rapid rule for predicting 30-day mortality and for managing geriatric patients with fever in the ED. The three factors are easy to memorize and apply in clinical practice. The Geriatric Fever Score can help emergency care, critical care, and geriatric care physicians decide on how best to manage geriatric patients with fever based on the urgency of their clinical condition. In addition, using the ratio of the actual to the expected number of deaths, this prediction rule may be used to evaluate the quality of geriatric care or of new clinical trials that include geriatric patients with fever. Despite the potential benefit of this score, it is well to remember that prognostic estimates are still only estimates. Providing geriatric medical care to patients always requires experienced clinical judgment and careful integration of objective data with other relevant information, such as the doctor-patient interaction and the patient's personal intentions and requirements.
